# The analysis of professional competencies of a lecturer in adult education

**DOI:** 10.1186/s40064-015-1014-7

**Published:** 2015-05-20

**Authors:** Iveta Žeravíková, Anna Tirpáková, Dagmar Markechová

**Affiliations:** CEIT Academy, Univerzitna 8431/6, 010 08 Žilina, Slovakia; Constantine the Philosopher University in Nitra, Andrej Hlinka 1, 949 74 Nitra, Slovakia

**Keywords:** Andragogy, Lecturer, Adult education, Mann-Whitney’ test, Wilcoxon’ test, Semantic differential method

## Abstract

In this article, we present the andragogical research project and evaluation of its results using nonparametric statistical methods and the semantic differential method. The presented research was realized in the years 2012–2013 in the dissertation of I. Žeravíková: Analysis of professional competencies of lecturer and creating his competence profile (Žeravíková 2013), and its purpose was based on the analysis of work activities of a lecturer to identify his most important professional competencies and to create a suggestion of competence profile of a lecturer in adult education.

## Introduction

In the years just after 1989, many companies with business activities focused on adult education started to work in Slovakia. At the same time, in Slovakia, a relatively large group of educators providing training for adults interested in this type of education in various fields appeared. After the initial euphoria on the side of participants, client organizations and educators themselves, the time of sobering came and variously motivated tendencies started to appear after the regulation of the Slovak adult education market. We can see the benefit of such a development of the market in the growing demand for quality and efficiency in education. In the 90s of the 20^th^ century, the question of the professionalization of lecturers’ work came into the centre of interest of the professional community in Slovakia. The job position of a lecturer still does not have an official status in Slovakia, it is at the level of a semi-profession. The identification of lecturers’ competencies, their description and standardization could contribute to establishing the job of a lecturer as a profession. That is why I. Žeravíková, the author of the dissertation Žeravíková 2013, undertook a research in 2012–2013 with the aim to identify lecturers’ most important professional competencies, based on an analysis of lecturers’ work activities in adult education. The notion of competence was used in the sense of one’s ability to apply knowledge in various situations (Prusáková 2005, p. 10). Competencies are based on activities, not only on the knowledge. They include communication, the development of the ability to learn, social competencies, problem solving, working with ICT, etc. It is evident, that professional competencies are closely related to the performance of a job. In the dissertation, the author worked with the definition of I. (Pirohová 2008, p. 20), who says: “The professional competencies of a lecturer can be characterized as the ability to give lectures influenced by the abilities, knowledge, experience and skills of a person, but also one’s willingness and capacity to use one’s potential functionally in adult teaching and to bear responsibility for one’s decisions during the educational process. Not every expert in a given field and profession has the same preconditions for performing the lecturer’s job. One’s qualities, the dimensions of one’s personality are important as well.” We understand the professional competencies as a complex demonstrated ability to perform certain professional activities; as a set of abilities and skills that enable one to perform a particular job – in our case it is the job of a lecturer in adult education.

The main research tool used during the realization of the research was a questionnaire, by means of which the author obtained information regarding the work activities of lecturers in adult education. For the statistical evaluation of the research results, nonparametric testing methods and the method of semantic differential were used. All calculations were done in EXCEL and STATISTICA.

The aim of the paper is to present the evaluation of the research results obtained by the use of semantic differential method and nonparametric methods of mathematical statistics and their interpretation.

### Characteristics of the research and the used methods

In the research, 287 respondents (159 lecturers and 128 adult learners) were involved. The questionnaire was the main research tool for conducting research. Two types of questionnaires were prepared and distributed:

A. Questionnaire to identify the work activities/tasks of lecturer in adult education.
B. Questionnaire for lecturers in the adult education and for participants of training.

The questionnaire A consisted of 55 questions and was addressed to the lecturers. The questionnaire B contained 61 questions and it was addressed to the lecturers and also to the participants of training. The questionnaires were distributed by the lecturer via e-mail (97 %) or personally (3 %) and their return was 96 %. The return of questionnaires for participants of training was 100 %, which was achieved by a personal requesting of the lecturer to complete the questionnaire immediately after completion of education. Lecturers were asked to complete both questionnaires and participants of training were asked to complete only the questionnaire B. In the questionnaires were used two notions: the real lecturer and the ideal lecturer. Category the real lecturer was the working designation of the actual state of the described situation. Category the ideal lecturer was the working designation for an ideal or a desired state in the going activities. The mentioned notions were explained to the respondents before completing the questionnaire.

The questionnaire A consisted of three parts. In the first part of the questionnaire, it has been detected whether the respondent pursues a lecturing activity of the lecturer. The next two sections of the questionnaire consisted of the section “a real lecturer” and the section “an ideal lecturer”. In the case that a respondent did not carry out lecturing activities, the respondent fills only the part “the ideal lecturer”, he did not fill the part “the real lecturer”. In the section called “the real lecturer” the respondents rated the activities that they are actually carried out. The evaluation scale was ranged from zero to six points, while six points were the best rating and zero points were the lowest rating.

The questionnaire B (addressed to the lecturers) was a reflection of the executive lecturers for their own activities with a focus on the competences of the lecturer. The questionnaire contained two parts, the part “real lecturer” and the part “ideal lecturer”. In the first part of the questionnaire, it was carried out to establish to what extent and in what quality lecturers actually carry out these activities and in the second part an idea to what extent and in what quality the lecturer should perform this activity ideally was evaluated. The evaluation scale was ranged from zero to six points, while the rating six points was the best rating and the weakest rating was zero points. Questionnaire B (addressed to the participants of training) investigated the view to the work of the real lecturer by the participants of training, who directly led the education and at the same time, the questionnaire investigated their idea about an ideal lecturer. The role of the respondents in the first part of the questionnaire was to express the extent to which and in what quality lecturers actually carry out these activities. In the second part of the questionnaire, the idea (by participants of training) about to what extent and what quality this activity lecturer should be perform in the ideal case was evaluated. The evaluation scale was identical to the range in the questionnaire addressed to the lecturer.

In the research was used the semantic differential method, whose authors are Osgood et al. 1957. Originally, this method was developed to measure the importance of the cognitive aspects later its use was expanded to humanities and social sciences in examining social perception, mental and social representations. Currently, the method of semantic differential is used also in a market research, in public opinion surveys, in advertising and in other areas. There are well known applications of this method for measuring the positions and various modifications of the original process are used. This method can be used also in pedagogy and psychology to explore how they differ the notions, for example, school year, holiday, signs, rewards, punishments, etc., in the semantic space of people. We can also compare how the different groups of respondents differ in the understanding of various concepts by the method of semantic differential.

### The source data and the basic numerical characteristics

Within the research, which we realized, we assessed the notions by the method of semantic differential: *real lecturer* (denoted *A*) and *ideal lecturer* (denoted *B*). The aim of the research was to compare the perceptions of the above notions in view of the participants of training and the lecturers. The semantic differential consisted of 61 six-point scales. In Table 1, the calculated average values for individual scales of the semantic differential are shown. The calculations were carried out in Excel. The table was complemented by column containing values ${d}_i^2$ (*d*_*i*_ is the average difference of values in *i*-th scale), which will be needed for further analysis of semantic differential data, and by a row of total sums.

**Table 1:** The average values for individual scales

Scale n. *i*	Lecturers			Participants of training		
	*A*	*B*	${d}_i^2$	*A*	*B*	${d}_i^2$
1	3.9	5.4	2.203	4.8	5.6	0.610
2	4.1	5.5	1.863	4.8	5.5	0.586
3	4.5	4.4	0.011	4.5	4.1	0.124
4	4.3	5.1	0.560	4.8	5.7	0.793
5	4.8	5.5	0.428	4.9	5.8	0.923
6	4.8	5.6	0.668	4.9	5.8	0.821
7	5.0	5.7	0.532	4.8	5.8	1.112
8	4.1	5.2	1.198	4.2	5.5	1.602
9	5.1	5.8	0.436	4.9	5.7	0.563
10	5.2	5.5	0.129	4.5	5.4	0.779
11	4.9	5.6	0.470	4.5	5.6	1.162
12	5.0	5.7	0.420	4.8	5.8	0.864
13	4.7	5.3	0.412	4.5	5.5	0.923
14	4.3	5.4	1.211	4.2	5.5	1.485
15	4.7	5.6	0.775	3.7	5.0	1.743
16	3.7	5.1	2.038	3.4	4.9	2.369
17	4.6	5.5	0.809	3.4	5.4	3.876
18	5.0	5.9	0.775	4.8	5.8	1.016
19	5.2	5.9	0.461	4.8	5.7	0.739
20	5.0	5.8	0.658	4.7	5.8	1.063
21	4.8	5.8	0.914	4.7	5.8	1.162
22	5.0	5.8	0.560	4.7	5.7	1.047
23	5.0	5.7	0.487	4.6	5.7	1.096
24	4.7	5.6	0.753	4.1	5.4	1.563
25	5.1	5.8	0.487	4.7	5.6	0.864
26	5.2	5.8	0.357	5.1	5.8	0.494
27	4.8	5.8	0.866	4.7	5.7	1.031
28	4.9	5.8	0.732	4.7	5.8	1.129
29	5.1	5.8	0.420	4.5	5.6	1.429
30	4.8	5.7	0.866	4.5	5.7	1.466
31	4.2	5.3	1.184	4.0	5.4	1.978
32	4.8	5.5	0.496	4.5	5.5	1.016
33	4.6	5.6	1.038	4.4	5.6	1.410
34	5.4	5.8	0.120	5.0	5.8	0.699
35	5.0	5.6	0.299	4.6	5.5	0.954
36	4.6	5.4	0.570	4.1	5.3	1.392
37	4.6	5.6	1.013	4.1	5.3	1.504
38	5.1	5.7	0.388	4.6	5.6	1.080
39	5.3	5.8	0.253	5.0	5.8	0.598
40	5.3	5.8	0.272	5.0	5.8	0.586
41	5.3	5.8	0.228	4.8	5.7	0.766
42	6.0	5.9	0.009	5.2	5.8	0.391
43	5.5	5.8	0.147	5.2	5.8	0.343
44	5.1	5.7	0.266	5.1	5.6	0.242
45	4.9	5.6	0.428	4.6	5.4	0.699
46	4.7	5.5	0.732	4.7	5.6	0.793
47	5.1	5.8	0.579	4.9	5.5	0.381
48	5.0	5.8	0.648	4.6	5.6	0.954
49	5.4	5.8	0.157	5.0	5.8	0.673
50	4.8	5.5	0.532	4.4	5.5	1.213
51	4.8	5.5	0.453	4.4	5.7	1.563
52	5.2	5.8	0.299	4.9	5.6	0.574
53	4.8	5.7	0.855	4.9	5.7	0.686
54	4.7	5.6	0.820	4.8	5.8	0.954
55	5.1	5.8	0.487	5.0	5.7	0.574
56	4.7	5.6	0.732	4.5	5.6	1.080
57	4.7	5.8	1.282	4.6	5.7	1.196
58	5.1	5.8	0.487	4.4	5.4	1.146
59	4.6	5.5	0.668	3.6	4.8	1.355
60	4.6	5.6	1.025	3.7	5.0	1.662
61	5.1	5.9	0.638	5.0	5.7	0.431
Σ	**296.60**	**342.65**	**39.607**	**279.90**	**339.34**	**63.328**

We calculated the numerical characteristics of semantic differential for both files, which are arithmetic averages (denoted ${\overline{x}}_A$ respective ${\overline{x}}_B$), dispersions (denoted ${\sigma}_A^2$ respective ${\sigma}_B^2$) and standard deviations (denoted *σ*_*A*_ respective *σ*_*B*_). The calculations were carried out in EXCEL. We obtained the following results:

1^st^ file - Lecturers:

$$ {\overline{x}}_A=4.86;\ {\overline{x}}_B=5.62;\ {\sigma}_A^2=0.15;\ {\sigma}_B^2=0.06;\ {\sigma}_A=0.392;\ {\sigma}_B=0.254 $$

2^nd^ file – Participants of training:

$$ {\overline{x}}_A=4.59;\ {\overline{x}}_B=5.56;\ {\sigma}_A^2=0.17;\ {\sigma}_B^2=0.09;\ {\sigma}_A=0.413;\ {\sigma}_B=0.294 $$

### The analysis of semantic differential data

For the statistical evaluation of the data of the semantic differential, we used the Mann–Whitney test, the Wilcoxon one-sample test, the statistics (Anděl 2003), *D*_*AB*_ (which characterizes the distance in the understanding the considered concepts) and the *Q*-correlation (Osgood et al. 1957). The calculations were carried out in EXCEL and STATISTICA.

#### Testing the statistical significance of differences in understanding the concept of a real lecturer by the considered respondent groups

First, we verify the assumption that the lecturers and participants of training perceive differently the notion of *a real lecturer* (notion *A*) by statistical test. The tested hypothesis is the null hypothesis *H*_0_ that between the considered groups of respondents there is no difference in the perception of the notion *A*. An alternative hypothesis is the hypothesis that lecturers and participants of the training perceive the given notion differently. Since it is not a justified assumption of a normal distribution, we tested the null hypothesis of a Mann–Whitney two-sample test. We chose the level of significance *α* = 0, 05. The calculations we made in the program STATISTICA. We received the following results in the computer output reports: the value of the testing criteria of a Mann–Whitney test is equal to 3.709914 and *p*-value is 0.0002073646900. The test was evaluated upon the computed value of the probability *p*. It is the probability of an error, which we commit if we reject the tested hypothesis. If this probability is lower than 0.05 respectively 0.01, we reject the tested hypothesis *H*_0_ at a significance level *α* = 0.05 respectively *α* = 0.01. Since the value of probability *p* is lower than 0.05, we reject the tested hypothesis at a significance level *α* = 0.05 in favor of the alternative hypothesis. Probability of error, which we have hereby committed, is almost zero. It was statistically proved that both lecturers and participants of the training perceive the considered notion *real lecturer* differently.

#### Testing the statistical significance of differences in understanding the concept of an ideal lecturer by the considered respondent groups

With the statistical tests we verified the assumption that lecturers and course participants perceive the notion of the ideal lecturer (notion *B*) differently. The tested hypothesis is the null hypothesis *H*_0_ that between the considered groups of respondents there is no difference in the perception of the notion *B*. An alternative hypothesis is the hypothesis that lecturers and participants of the training perceive the notion *B* differently. Since it is not a justified assumption of normal distribution, we tested the null hypothesis of a Mann–Whitney two-sample test. We chose the level of significance *α* = 0, 05. The calculations we made in the program STATISTICA. We received the following results: the value of the testing criteria of a Mann–Whitney test is equal to 0.2278246386 and *p*-value is 0.2278246386. Since the value of probability *p* is greater than 0.05, we cannot reject the tested hypothesis *H*_0_. That means that the observed differences between the considered groups of respondents are not statistically significant in the perception of the notion *B*.

#### Testing the statistical significance of differences in understanding the concepts of a real lecturer and an ideal lecturer by the considered participants of training

Next, we will be interested in whether the participants of training perceive the notions of a real lecturer and an ideal lecturer differently. The tested hypothesis is the null hypothesis *H*_0_ that the participants of training perceive the notions *A* and *B* equally. An alternative hypothesis is the hypothesis that the participants of training perceive the notions *A* and *B* differently. We tested the null hypothesis *H*_0_ with the help of a Wilcoxon one sample test. The calculations we made in the program STATISTICA. We received the following results in the computer output reports: the value of the testing criteria of Wilcoxon one-sample test is equal to 6.784132 and *p*-value is 0.000000. Since the *p*-value is approximately zero (approximately because this number is rounded to six decimal places), we rejected the tested hypothesis at the arbitrarily small level of significance. Probability of error, which we have hereby committed, is almost zero. Thereby it has been statistically proven that the participants of training perceive the notions of *a real lecturer* and an *ideal lecturer* differently.

#### Testing the statistical significance of differences in understanding the concepts of a real lecturer and an ideal lecturer by the considered lecturers

In the following, we will be interested in whether the lecturers perceive differently the notions of a *real lecturer* and an *ideal lecturer*. The tested hypothesis is the null hypothesis *H*_0_ that the lecturers perceive the notions *A* and *B* equally and the alternative hypothesis is the hypothesis that the lecturers perceive the notions *A* and *B* differently. We tested the null hypothesis *H*_0_ with the help of a Wilcoxon one-sample test. The calculations we made in the program STATISTICA. We received the following results in the computer output reports: the value of the testing criteria of Wilcoxon one-sample test is equal to 6.769766 and *p*-value is 0.000000. Since the *p*-value is approximately zero, we rejected the tested hypothesis at the arbitrarily small level of significance. Probability of error, which we have hereby committed, is almost zero. Thereby it has been statistically proven that lecturers perceive the notions of *a real lecturer* and an *ideal lecturer* differently.

#### Determination of the distance between the concepts

The distance between the concepts *A*, *B* can be assessed by the statistics *D*_*AB*_, which is defined by the formula ${D}_{AB}=\sqrt{{\displaystyle \sum_{i=1}^k{d}_i^2}},$ where *d*_*i*_ is the difference of the average values in the *i*-th scale (Reiterová 2003). Statistics *D*_*AB*_ is a simple measure, which represents the linear distance between concepts *A*, *B*. The lower is the value of statistics *D*_*AB*_, the smaller is the distance between notions *A*, *B*. On the contrary, higher value of statistics *D*_*AB*_ means bigger distance between notions *A*, *B*. Due to the result of Wilcoxon test, it makes sense calculate the distance between considered notions A, B for the individual files of respondents. Use the data in Table 1 we calculated the following statistics values:

1^st^ file - Lecturers:

$$ {D}_{AB}=\sqrt{{\displaystyle \sum_{i=1}^k{d}_i^2}}=\sqrt{39.607}=6.293 $$

2^nd^ file – Participants of training:

$$ {D}_{AB}=\sqrt{{\displaystyle \sum_{i=1}^k{d}_i^2}}=\sqrt{63.328}=7.958 $$

Based on the calculated values of the statistics *D*_*AB*_ we can see that the greater distance of the concepts of a real lecturer and an ideal lecturer is for the participants of training. As could be expected, the participants of training evaluated the work of lecturers more critically than the lecturers themselves.

### *Q*-correlation

Besides the statistics *D*_*AB*_ the semantic differential data can also be analyzed with the help of *Q*-correlation, which is a modification of the multiplicative correlation and expresses the degree of similarity between two profiles. According to the degree of similarity of profiles, the similarity of concept understanding can be assumed. *Q*-correlation is described by the correlation coefficient *Q*_*AB*_, which is defined by the formula

$$ {Q}_{AB}=1-\frac{{{\displaystyle \sum_{i=1}^k{d}_i^2-k{\left({\overline{x}}_A-{\overline{x}}_B\right)}^2-\left({\sigma}_A-{\sigma}_B\right)}}^2}{2k\cdot {\sigma}_A\cdot {\sigma}_B}. $$

The coefficient *Q*_*AB*_ assumes its values in the interval 〈 − 1, 1〉. These values are interpreted in the same way as the values of Pearson’s correlation coefficient. The value 1 means complete consistency in understanding the notions *A*, *B*, the value −1 means completely contradictory understanding of notions. Zero value of the coefficient *Q*_*AB*_ means zero consistency in understanding notions *A*, *B*. Higher absolute value means closer dependence (direct or indirect) in the understanding of notions *A*, *B*.

In the following, we will calculate the values of coefficient *Q*_*AB*_ for the considered pairs of respondents.

1^st^ file - Lecturers:

$$ {Q}_{AB}=1-\frac{39.607-61\cdot {\left(4.86-5.62\right)}^2-{\left(0.392-0.254\right)}^2}{2\cdot 61\cdot 0.392\cdot 0.254} = 0.6415 $$

2^nd^ file – Participants of training:

$$ {Q}_{AB}=1-\frac{63.328-61\cdot {\left(4.59-5.56\right)}^2-{\left(0.413-0.294\right)}^2}{2\cdot 61\cdot 0.413\cdot 0.294} = 0.6004 $$

Because the goals of andragogy are included in the status “an ideal lecturer”, the Q-correlation can be considered as the measure of dependence between lecturer’s competences and the goals of andragogy. Based on the calculated values of the correlation coefficient (= 0.6004), we can consider about a moderate degree of conformity between lecturer’s competencies and the goals of andragogy.

Based on the calculated values of the coefficient *Q*_*AB*_, we see further that in the perception of the notions of a real lecturer and an ideal lecturer by the participants of training is somewhat less consensus as in the perception of the considered notions by lecturers. This means that the participants of training evaluated the work of lecturers more critically than the lecturers themselves. However, the difference is not very significant. Therefore, we will further analyze the above notions in each of the dimensions of the semantic differential.

#### Analysis of the data of semantic differential under each of the dimensions

The individual adjectives of semantic differential can be divided according to the dimensions. Osgood divided the individual adjectives into three dimensions, while in each dimension was the same number of adjectives. According to other authors (e.g., Kerlinger 1972) it is not necessary to select the three dimensions, and the dimensions do not need to consist of an equal number of adjectives. However, it is important that adjectives were relevant to the notion. Since in the identification of lecturer’s professional competence we used a model of competence by Prusáková, 2005 we divided the scales of semantic differential into three dimensions:

- dimension A – we included the professional andragogical competence of a lecturer into this dimension;
- dimension B – we included social competence of a lecturer into this dimension;
- dimension C – we included cognitive competence lecturer into this dimension.

The dimension A consisted of 17 scales, dimension B of the 32 scales and dimension C of the 12 scales. The subject of further analysis will be the comparison of how the analyzed notions are perceived by the given groups of respondents within individual dimensions. We calculated the values of the numerical characteristics *D*_*AB*_ and *Q*_*AB*_ for individual dimensions. We obtained the following results:

dimension A

1^st^ file - Lecturers:

$$ {\overline{x}}_A=4.56;\ {\overline{x}}_B=5.39;\ {\sigma}_A^2=0.18;\ {\sigma}_B^2=0.10;{\sigma}_A=0.42;\ {\sigma}_A=0.42;\ {\sigma}_B=0.32;\ {D}_{AB}=3.763;\kern0.5em {Q}_{AB}=0.466 $$

2^nd^ file – Participants of training:

$$ {\overline{x}}_A=4.45;\ {\overline{x}}_B=5.45;\ {\overline{x}}_B=5.45;\ {\sigma}_A^2=0.24;\ {\sigma}_B^2=0.17;\ {\sigma}_A=0.49;\ {\sigma}_B=0.41;\ {D}_{AB}=4.510;{Q}_{AB}=0.512 $$

dimension B

1^st^ file - Lecturers:

$$ {\overline{x}}_A=5.02;\ {\overline{x}}_B=5.72;\ {\sigma}_A^2=0.11;\ {\sigma}_B^2=0.02;\ {\sigma}_A=0.33;\ {\sigma}_B=0.15;\ {D}_{AB}=4.143;\ {Q}_{AB}=0.{5412}^{\mathrm{n}} $$

2^nd^ file – Participants of training:

$$ {\overline{x}}_A=4.69;\ {\overline{x}}_B=5.64;\ {\sigma}_A^2=0.10;\ {\sigma}_B^2=0.03;\ {\sigma}_A=0.32;\ {\sigma}_B=0.16;\ {D}_{AB}=5.528;\ {Q}_{AB}=0.495 $$

dimension C

1^st^ file - Lecturers:

$$ {\overline{x}}_A=4.86;\ {\overline{x}}_B=5.67;\ {\sigma}_A^2=0.04;\ {\sigma}_B^2=0.02;\ {\sigma}_A=0.21;\ {\sigma}_B=0.15;\ {D}_{AB}=2.877;\ {Q}_{AB}=0.471 $$

2^nd^ file – Participants of training:

$$ {\overline{x}}_A=4.51;\ {\overline{x}}_B=5.51;\ {\sigma}_A^2=0.20;\ {\sigma}_B^2=0.09;\ {\sigma}_A=0.45;\ {\sigma}_B=0.30;\ {D}_{AB}=3.526;\ {Q}_{AB}=0.8734 $$

From the data analysis of the semantic differential within individual dimensions, it follows that participants of training see the greatest consistency between competencies of a real lecturer and an ideal lecturer in cognitive competencies of the lecturer. Since the content of cognitive competencies is knowledge and competence about the basics of cognitive processes, about psychological and physiological functioning of human brain essentially, about acquisition, processing and transmission of information, these competencies are difficult to see in learning process directly.

We presume that the problems regarding the objectiveness of recipients’ evaluation could be the explanation of the correspondence between the competencies of a real and an ideal lecturer as perceived by training participants.

Similarly, the lecturers evaluated their own cognitive competencies as satisfactory; according to them, the smallest difference between the competencies of a real and an ideal lecturer could be found in the field of cognitive competencies. This can be caused by the interdependence between the cognitive competencies and the expertise of a lecturer. The expertise of a lecturer is understood as a set of professional skills developed during their education in the field of practice of the particular lecturer (e.g. technical sciences, law, economics, natural sciences, etc.). Considering the content of competencies and the achieved qualification, lecturers may experience balance between the competencies of a real lecturer (themselves), and of an ideal lecturer. In spite of the above mentioned, this result does not allow us to conclude that there is such a high level of competencies in this field that we could consider it to be the ideal state of affairs. Moreover, the ideal state of affairs has not even been defined.

Based on the analysis of semantic differential data within individual dimensions we can state, that participants of training see the biggest differences between the competencies of a real and an ideal lecturer in the field of lecturer’s social competencies. On the contrary, according to the lecturers, the highest correspondence between the competencies of a real and an ideal lecturer can be observed in the field of social competencies.

#### Further analysis

Considering the results of the analysis of semantic differential data within individual dimensions, we were also dealing with the influence of other aspects on the social competencies of a lecturer. We analyzed the impact of lecturer’s field of work (law, economics, natural sciences, technical sciences, information technologies, humanities, languages, etc.), qualification (technical, legal, medical, pharmacological education, etc.), form of employment (an internal or external lecturer) and length of practice on their social competencies.

By means of statistical methods it was proven, that the lecturers’ field of practice has statistically significant impact on their social competencies. Social competencies are part of a lecturer’s competency profile, which means that the lecturer’s field of practice has an immediate impact on the lecturer’s competency profile. We found out that lecturers working in the field of humanities dedicate significantly more time to activities requiring social competencies than the lecturers working in other fields being identified. The percentual differences between the individual fields of practice indicate that lecturers working in the field of humanities and languages perform more activities related to social competencies than lecturers working in other fields do. We can assume that it can be a matter of a natural inclination of lecturers working in these fields.

We also found out that the length of practice has a statistically significant effect on lecturers’ activities in the field of their social competencies. As much as 100 % of the respondents working as lecturers for 1 to 3 years – spend, on average, a lot of time by doing activities based on social competencies. As well as lecturers with different length of their practice indicated high amount of time (five or six – a lot of time, respectively an extreme amount of time) spent by activities requiring social competencies. The findings document a statistically significant dependence between the length of lecturer’s practice and activities, which indicate social competencies. Social competencies are developed in relation to the personal traits of lecturers, but they can be acquired gradually through lecturers’ work or further education as well. For lecturers, it is, of course, useful to have a list of competencies at the beginning of their professional career because it can make their work with individual participants of education and with whole groups easier. As they can be developed gradually, their partial deficiency at the beginning of the lecturers’ practice does not necessarily have to be a fundamental problem.

Our results show that the form of employment (internal or external) has a statistically significant impact on the activities of lecturers in the field of their social competencies. Activities related to social competencies are influenced by the lecturers’ form of employment. It can be explained by the fact that external employees are not necessarily closely linked to their organization. As they are not internal employees, they are less sensitive to the relationships within the organization and to the relationships with the participants of education. External lecturers may, on one hand, keep distance and have a detachment regarding the above-mentioned relationships, on the other hand, they do not have to recognize nor to perceive the possible problems inside the organization. All these circumstances can influence their activities related to social competencies.

It was proven that the achieved qualification significantly influences the activities of a lecturer in the field of social competencies, too. Our findings show that

- lecturers‘field of practice has a significant impact on their social competencies and is closely linked to their professional qualification,
- length of lecturers’practice has a statistically significant effect on their activities in the field of social competencies,
- internal or external form of employment has a significant impact on the lecturers’activities in the field of social competencies,
- achieved qualification significantly influences lecturers’activities in the field of social competencies.

These findings could be the indicators of the results of the analysis of semantic differential data within individual dimensions, which demonstrate that the biggest differences between the competencies of a real and an ideal lecturer are perceived by the course participants in the field of lecturers’social competencies. As the field of social competencies is based on competencies such as communication, cooperation, facilitation, feedback, etc., it is a part of those lecturers’ activities, which are visible in the educational process and where the lecturers’ professionalism can be registered. This is the field where the participants can identify – e.g. through nonverbal communication – the signals that accompany the work of a lecturer in a positive or a negative sense. Lecturers can be evaluated by the participants of education best in a direct interaction. The participants of education perceive some discrepancy between a real and an ideal lecturer in the field of social competencies. It could be explained by a natural tendency to be more critical when assessing the performance of other people as well as by the objective state of being of the perceived and evaluated reality. These presumptions were proven by the observed correspondence between the competencies of a real and an ideal lecturer in lecturers’ responses. It could be caused by their positive self-image and self-evaluation, which do not correspond with the reality. In spite of that, we do not intend to challenge the work of many lecturers, who are real professionals in their field of practice.

## Conclusions

The study presents the results of a research project from the field of andragogical research that was realized in Slovakia in 2012–2013 as a part of a dissertation (Žeravíková 2013). In the analysis of a selected set of lecturers’ professional competencies – especially the social competencies – we point out their importance in lecturers’ work. Social competencies, together with instructional competences, andragogical, cognitive and personal competencies form the competency profile of a lecturer. The need for dealing with the issues of lecturers’ professional competencies arises from the situation in adult education in Slovakia on one hand, and the requirements of the andragogical theory on the other hand. The demand for the identification and the analysis of professional competencies comes from Slovak organizations, research institutes of Slovak universities, and the bodies of state administration in Slovakia and the EU. This situation is influenced by the prevailing trend to deal with the issues of education and training of lecturers in the context of their competencies and competency profile. The main objective of these attempts is to get lecturers’ training to a certain level in accordance with the quality requirements. Lecturing in Slovakia has gradually been gaining the features, characteristics and activities typical of a profession. We see this as the process of professionalization of lecturers’ job, which is a precondition for becoming a profession. If we want the job of a lecturer to be considered a profession, it must fulfill several criteria. For fulfilling them, we find it very important to prepare a set of competencies necessary for practicing this profession, to standardize the performance of lecturers’ activities and to establish professional qualification norms. In the process of identification of lecturers’ professional competencies, we worked with Prusáková’s model of competencies (Prusáková 2005), which includes the following three groups of competencies:

- professional andragogical competencies,
- social competencies,
- cognitive, respectively intellectual competencies.

We compared Prusáková’s model with the results of our research. Based on the research results, we designed a competency profile of lecturers in adult education. We suggest to divide the competencies into the following five basic groups.

- social competencies,
- cognitive competencies,
- instructional competences,
- personal competencies,
- andragogical competencies.

The order of the listed competencies does not reflect their importance. Next, we are going to characterize the above-mentioned groups of competencies.

Social competencies should include knowledge (and the consequent capabilities) on communication as a verbal and nonverbal process, its components, contexts, communication styles, communication techniques, active listening, and knowledge on providing and receiving feedback, on education as a communication process. In addition, knowledge on working with a group of students, managing group dynamics, principles of cooperation, facilitation, motivation, etc. should be included.

Cognitive competencies should include knowledge (and the consequent capabilities) on the basic cognitive processes, psychological and physiological principles of human brain functioning, acquisition, processing and the transfer of information, etc.

In the case of cognitive competences, it is important to realize that lecturers are adult learners at the same time. Lecturer’s cognitive competences are the presuppositions of lecturing activity on one hand, but they are the basis for the achievement of learning objectives on the other hand.

Lecturer’s experience is a valuable source of learning as well. However, we should be aware of the fact that primarily a lecturer-activist learns from experience when applying P. Honey and A. Mumford’s typology and their model of learning styles created in 1992 (Honey and Mumford 1992). Strong activists go about new experiences full-face and broad-minded. They are open to different opinions and usually inspired by anything new. The other types – a theoretician, a reflexive (thoughtful) type and a pragmatist prefer other ways of learning. The thoughtful type likes considering experience from afar and looks at it from various points of view. He/she collects data and studies them thoroughly before making any conclusions. The theoretician accepts information and includes them into logical theories. He/she analyzes problems gradually and logically. He concludes the facts into coherent theories. The pragmatist likes to test, whether the ideas, theories and techniques work in practice. He/she searches new ideas and makes experiments with their application. He/she reacts to a problem as to stimulation.

We realize that the existence of specific unilateral type of a lecturer, as we have briefly mentioned above, would cause problems in lecturers’ work. Therefore finding the lecturer’s inclination to a specific type is more interesting, mainly when we realize the benefits and risks of such inclination. Each lecturer should know his/her typical methods of learning which are significant for his/her inclination to specific methods of training. Lecturer should be aware of such inclinations and work with them so that his/her lecturing activity will be effective for the participants of training. We are sure that the experience as a source of learning is an inevitable condition of successful lecturer’s work. The fact, that some types of lecturers learn from experience “naturally” and “easily” while the other types of lecturers have to strive more cognitively to exploit experience as a source of learning, is equally important. Lecturer’s ability to apply experience as a source of learning is his/her individual advantage.

Instructional competences should include knowledge (and the consequent capabilities) on didactic theory of adult education, including the goals of education, creating a positive learning atmosphere, structure of the educational unit, monitoring and assessment of the outcomes of education, instructional principles, forms, methods, application of teaching tools and technologies, teaching materials, etc.

The ability of lecturer to apply the educational objectives into case studies or work-related problems and to search for opportunities of such application within the education process is a very important instructional competence. This kind of application is the most suitable method of learning for an adult learner. The adult learners’ knowledge can be both acquired and remembered effectively via their experience in certain situations. Education of adults without application of case studies and without application of objectives into work-related problems has very low effect and minimum success not only in client companies (buyers of education), but also among individual participants of training. Thus, the instructional competence becomes one of the basic capabilities of lecturers and namely their “strategic” skill if they can apply it effectively in their lecturing activity.

Personal competencies should include knowledge (and the consequent capabilities) on self-evaluation, self-regulation, self-knowledge and self-development. Personal competences also include self-directed learning. If lecturers should stand up in lecturing activity and graduate professionally, they have to learn permanently and work on their personal development. Deficiency in lecturers’ personal competencies will be eventually revealed by certain signals. Participants perceive such signals sensitively and are able to decode them. Lecturing activity of such lecturers will be temporary and may be problematic.

And lastly, andragogical competencies should include knowledge (and the consequent capabilities) on adult education (birth, development, current state of adult education, basic terminology, andragogy as a science, the functions and the structure of andragogy, institutionalization and organization of adult education, adults in andragogy, the target groups of adult education, professionalization of andragogical work).

As the mentioned characteristics are an expression of our subjective view on the given field, we offer the content of individual competencies for discussion to the professional community. At the same time, we realize that the borders between individual competences are very vague and loose in some cases, and that some lecturers’ capabilities, e.g. self-directed learning or using experience as a source of learning, influence lecturers’ instructional competences as well.

By means of this study, we intended to contribute to the attempts to professionalize lecturers’work in Slovakia, especially to standardization of lecturers’competencies required for practicing this profession in such a way that a system quality evaluation tool of lecturers’work, which would include the qualification standards as well as the evaluation standards, could be designed.
